# Investigating parental perspectives of the enablers and barriers to communication with their preterm infants: A narrative study

**DOI:** 10.1177/13674935241302437

**Published:** 2024-11-26

**Authors:** Julia Petty, Celia Harding, Lisa Whiting

**Affiliations:** 1 School of Health and Social Work, 229434 University of Hertfordshire, Hatfield, UK; 2 Division of Language and Communication Science, City University of London, London, UK; 3 City St. George's, University of London, London, UK

**Keywords:** Communication, family, narrative, neonatal, parenting support

## Abstract

Learning to communicate with infants in a neonatal unit setting is challenging. Parents need time and support to feel confident and acquire skills that enable them to care for, be close to, and communicate with their infant. This qualitative, narrative-based study sought to investigate parents’ understanding of factors that enhance or prevent the development of early communication and interaction between preterm infants and parents within a neonatal setting. Our study used a narrative interview approach with eight parents of premature infants, to explore the enablers and challenges to communication. Reflexive thematic analysis revealed four main themes: Impact of being in the neonatal unit, different communication strategies, communication barriers and an ongoing need for support at home. Our findings provide parental insight into communication between themselves and their premature infants. Overall, parents spoke highly of communication strategies that they were taught but it was clear they received varying advice and support, in the neonatal unit and post-discharge. There is a need for clear, consistent, and culturally appropriate communication strategies with greater awareness of how to facilitate them. Since failure to enable parent–infant interactions may potentially mean delayed language development, there is an essential need for tailored parent-accessible resources.

## Introduction

Learning to communicate with infants in a neonatal unit setting is challenging and parents need time and support to feel confident and acquire skills that enable them to care for and be close to their infant (Evans et al., 2014). This is particularly important given the stress imposed on parents by the admission of their infant to neonatal care (Petty et al., 2020). Involvement in care can promote physical and emotional closeness for both infants and carer-givers creating important precursor skills that facilitate parent–infant interaction and communication (Flacking et al., 2012).

Despite many perceived benefits of language and communication stimulation for preterm infants, vital approaches including bonding, attachment, and skin–to-skin care are often incorrectly referred to in the literature as ‘communication’ (Harding et al., 2022). Although these approaches are necessary for facilitating productive dyadic relationships, guidance for developing ways of interacting to support linguistic skills is inconsistent (Harding et al., 2019, 2022) – this includes use of facial expression, gesture, tone of voice, responsiveness to vocalisations, appropriate use of vocabulary and developing receptive language abilities. Few studies have investigated the impact of positive communication on parents despite speech, language and communication difficulties being a known developmental risk for infants born preterm (Harding et al., 2019).

### Preterm birth and associated speech, language, and communication risks

Speech, language, and communication (SLC) difficulties can impact on childhood learning and development within school and home settings and such delays or disorders can impair development of social learning and interaction and inhibit inclusion (Johnson et al., 2015). Children born preterm are at higher risk of developing speech, language, and communication difficulties (Stene-Larsen et al., 2014); cohort studies of infants born preterm have shown that a lower gestational birth age can increase SLC difficulties (Zambrana et al., 2021). Other studies also identify a risk of substantive and wide ranging SLC challenges and delays in groups of children born preterm with later gestational birth ages (Sansavini et al., 2021; Gayraud and Kern, 2007; Kisilevsky et al., 2009; Kuzniewicz et al., 2014).

Specific SLC problems include less use of gesture and pointing (Benassi et al., 2018), difficulties developing the ability to link words effectively, delayed acquisition of first words, difficulties with grammatical rules and conversations, shorter sentences and fewer nouns and verbs within expressive language repertoires (Imgrund et al., 2022; Sanchez et al., 2019). Aside from expressive language deficits, children born preterm are also at risk of receptive language (understanding of language) difficulties which can impact on the acquisition of other cognitive skills (van Noort-van Der Spek et al., 2012). Those preterm infants who are likely to have receptive language problems may develop shorter mean length of expressive utterances at 2 years, with persisting problems still showing at 4 years (Jansson-Verkasalo et al., 2004).

### Neonatal environment and speech, language, and communication

Noise levels within neonatal units can increase an infant’s energy consumption, lead to physiological instability and impact on their hearing (Séassau et al., 2023). However, consistent use of familiar voices, including voice modulation through talking or reading aloud, can calm an infant and allow physiological stable states to emerge (Saliba et al., 2018). On-going use of spoken language by parents, directed towards their preterm infant in a neonatal setting, promotes more infant vocalisations and better language and cognitive outcomes at 7 and 18 months (Caskey et al., 2014).

Parent stress behaviours can inhibit positive interaction, but when supported to provide specific communication skills within a neonatal unit, mothers are more responsive to their infant’s cues and show fewer stress behaviours (Milgrom et al., 2013); benefits from receiving direction as to how to communicate with an infant when on a neonatal unit is maintained by families 6 months post-discharge (Milgrom et al., 2013). Direct support that specifically targets communication can help parents to use relevant strategies such as gaze, eye-contact and meaningful interactions with infants (Benassi et al., 2018; Caskey et al., 2014; Hilbrink et al., 2015). Parents rate guidance to encourage interaction with their infant as important, especially during feeding; professional support that guides families to develop interaction is rated as being helpful (Guillaume et al., 2013).

Nurses are more likely to offer emotional and practical support for attachment and parent empowerment, rather than directing carers to use specific strategies to develop communication (Maleki et al., 2022). Within the literature, communication is still misunderstood and misrepresented; this is because research investigating it confuses parent and neonatal staff dynamics with staff use of language with infants (Guillaume et al., 2013).

### Improving infant–carer communication

Harding et al. (2019, 2022) highlight that speech, language and communication development, as well as support to encourage and promote essential building blocks to support later linguistic development between parents and infants, needs to be better explained and understood. Given that infants born preterm are at higher risk of developing SLC problems and disorders, it is imperative that communication is recognised beyond interpretation of infant states and homeostatic regulation, and that communication and interaction is integrated more effectively into neonatal care so that positive dyadic synchrony can be established as an infant develops (Harding et al., 2022). Strategies such as bonding, attachment, responsiveness to cues and skin-to-skin care are essential preparatory skills for development of productive dyadic relationships, but these approaches alone do not lead to meaningful interaction (Harding et al., 2022). Infants are socially predisposed to seek social interaction with others from birth, to gaze at a human face and develop turn-taking as a precursor to early conversations, and this body of knowledge needs to be adopted into communication programmes (Dominguez et al., 2016; Hilbrink et al., 2015). Healthcare professionals, in particular speech and language therapists, have an important role in providing communication support within a neonatal unit so that sustained synchronous development between parents and infants is sustained (Harding et al., 2022).

## Aim

To investigate parents’ understanding of factors that enhance or prevent the development of early communication and interaction between preterm infants and parents within a neonatal unit environment.

## Methods

### Study design

We employed a qualitative, interpretive, interview design which is suited to research that aims to explore experience and views (Holloway and Galvin, 2016), in this case, factors that enhance and hinder communication with preterm infants on a Level 1 and 2 neonatal unit in a United Kingdom [UK] city district. Within this design, narrative inquiry was the chosen methodological approach as this focuses on an individual’s experience which is expressed through stories, spoken, or written accounts of connected events (McDrury and Alterio, 2016). Narrative inquiry focusses on perceived experiences of individuals and how their physical, social, and cultural environment impacts and shapes the narratives they tell (Haydon et al., 2018).

A Parent Advisory Group, comprising three parents from a neonatal/parent support charity, was established. They were consulted about the participant information sheet, consent form, interview schedule, and support information sheet.

### Population and recruitment

Recruitment of parents was via purposive sampling; potential participants received study information, via posters on the walls of the parents’ room at both neonatal unit settings. Inclusion criteria were parents who:

- Had a premature infant born before 37 weeks gestation (this could include a multiple birth).
- Had spent more than a week in a neonatal unit before discharge home.
- Were fluent in the English language.

Parents were excluded if they had a very sick infant with an uncertain outcome and/or a infant with a complex or congenital condition (as determined by our gatekeepers).

Identified gatekeepers (a neonatal consultant and nurse manager/educator) acted as conduits of information between our research team and participants. As well as recruitment posters, gatekeepers approached potential participants (at appropriate opportunities) to explain the study and ascertain potential interest.

### Ethical Considerations

We received Health Research Authority approval (protocol number SB\LG\101010662\721189).

Informed consent was obtained from participants prior to participation and data collection. All interview transcripts were assigned a code and data de-identified. Written records of participants’ names and consent forms were kept separately from research data and were stored in a secure OneDrive folder with password protected access at a university establishment. The Standards for Reporting Qualitative Research (O’Brien et al., 2014) were used to guide our study protocol.

### Data collection

Narrative interviewing was employed; this is an unstructured approach often selected by researchers wishing to generate qualitative data (Peters and Halcomb, 2015). It encourages interviewees to talk freely (Elliott, 2005) about their experiences, starting with one main question (Spector-Mersel, 2010) that is intentionally broad as it aims to encourage participants to construct their own narrative. In a healthcare context, narrative interviewing is used to collect participant stories about health/illness experiences – in our case, this was early infant communication within a neonatal unit. This approach enables each participant to have control over pace, direction, and interview content (Anderson and Kirkpatrick, 2016) without any element of formality whilst still permitting the interview to be guided by the research aim (Holloway and Galvin, 2016).

One researcher [JP], who had previous experience of narrative inquiry, undertook all the interviews, was unknown to the parents and had no previous employment with either of the neonatal units. A topic guide, informed by existing literature, was developed to fulfil the research aim; this was used to prompt as needed; each parent participated in one interview (there was no data collection with couples). Interviews were conducted between November 2022 and February 2023, via videoconferencing and were digitally audio recorded. Audio-recordings were sent securely to a professional transcriber to convert them verbatim to text-based transcripts.

### Analysis

Narrative thematic analysis (Holloway and Galvin, 2016) was used to capture and describe parents’ experiences and perceptions consistent in part with an approach postulated by Riessman (2008) and a hybrid approach by Floersch et al. (2010). Analysis was undertaken by LW; an inductive approach, using Microsoft Word, was used. Interview transcripts were read several times to acquire an overall sense of meaning; significant and meaningful statements were captured within the transcripts through allocations of initial codes. Each code provided a concise label of the core element of the extracted statement. Individual codes were then organised into preliminary categories by exploring similarities and differences within each participant’s description (Floersch et al., 2010). Again, proposed themes were reviewed by another, different member of the research team, and were refined before we came to a consensus on the confirmation and naming of the final themes.

## Findings

Eight parents (all mothers) participated in the narrative interviews, which lasted between 37 and 72 minutes; parent participant characteristics are summarised in Table 1.

**Table 1.** Participant details.

Parent (*p*)	Age range (years)	Ethnicity (described by the participant)	Gestation of baby at birth (in weeks and days)	Sex of baby	Weight at birth	Length of time in neonatal unit	Age of baby at time of interview	Number of other children
Fatima (F)	41–45	British Asian Indian	27	M	1.065 Kg	3 months	10 months	1
Sharon (F)	36–40	Black British	24	F	1.1875 Kg	3 months	9 months	1
Krystyna (F)	36–40	White Polish	31	M	1.3 Kg	6 weeks	12 months	1
Charlotte (F)	30–35	White British	24	M	0.690 Kg	4 months	11.5 months	1
Maryam (F)	30–35	British Asian Indian	28 + 6	F	1.35 Kg	8 weeks	3.5 months	1
Sue (F)	30–35	White British	29 + 1	M	1.275 Kg	11 weeks	12 months	0
Jessica (F)	30–35	White British	34	M	2.268 Kg	4 weeks	5 months	1
Aiyla (F)	26–30	Turkish	29 + 5	M (twins)	1 kg and 1.2 kg	8 weeks	8 months	0

F = Female; M = Male.

Data analysis revealed four themes and these are presented below with the titles providing our professional interpretation as well as an illustrative parental quote. Pseudonyms have been used throughout.

## Impact of being in a neonatal unit: *‘So overwhelmed by everything’* [Charlotte]

Being admitted to a neonatal unit, posed a new and challenging experience, particularly since parents had no previous contact with premature infants and/or neonatal units. This exposed them to a life changing event involving many technologically based interventions as well as health professionals who were focused on providing immediate and often emergency care for their infant. As a consequence, communication was not felt to be an immediate priority; furthermore, three parents initially felt that this was made more difficult because they perceived that their:

‘baby was the hospital’s property’ [Sharon]

These parents spoke about needing permission to, for example, open an incubator door, but also receiving mixed messages from nursing staff in relation to situations such as this.

Support and advice in relation to communication came from many sources (lactation consultants, psychoanalysts, occupational therapists, behavioural and speech and language therapists), but the personnel and type of support/advice varied from one neonatal unit to another. When expert advice was available, this was spoken of very positively:

‘So very quickly you are introduced to lactation consultants and speech and language therapists…she [behavioural therapist] was very good at talking to us about just what his body language was’. [Fatima]

‘I think the…it must have been the occupational therapist that talked about sort of speaking in low tones and how to communicate better’. [Maryam]

‘The nurses were very helpful showing us how to do the skin-to-skin, how to talk to the baby…even the baby is sleeping we can still touch him, talk to him’. [Krystyna]

One parent realised that an endotracheal tube meant that they were unable to hear their infant cry so were taught how to recognise behavioural cues. Whilst the parents had considerable contact with nursing staff, their advice and encouragement in relation to communication was broader and primarily focused on handling of the infant and activities such as skin-to-skin contact (which all eight parents spoke of favourably).

## Different communication strategies: ‘*I was doing all of that like a way of communicating*’ [Aiyla]

Parents spoke about a range of strategies, including singing and talking, that they used to communicate with their infant and how these:

‘Made the bond with us’ [Krystyna]

On one occasion, a parent talked about the ‘*lovely*’ [Sharon] singing therapy that she and her infant had experienced in a children’s intensive care unit (rather than a neonatal unit); the therapist sung the infant’s name accompanied by a guitar. Other parents used song themselves:

‘I would sort of talk and sing’ [Maryam]

‘So I would do that every day, sing to them [twins]’ [Aiyla]

Participants also valued touch, feeling that this enhanced their communication and helped to build a bond with their infant. This included spontaneous touch as well as giving care (such as nappy changing) and skin-to-skin contact:

‘Definitely the skin-to-skin, like 100%, I thought it was brilliant and I think my mental health improved as well, just holding her, touching her, smelling her’. [Sharon]

‘Oh we did it [skin-to-skin] with him, yeah, for hours and hours and hours, I swear it made him better the quickest’. [Sue]

A cuddle, particularly the first one, was a cherished and exciting moment although, for some, this type of physical contact was delayed because of how sick their infant was and that was ‘*really difficult*’ [Sharon]. Parents also learnt the best times of the day to communicate with their infant, understanding when they were more alert and receptive.

Despite a desire to build a parent–infant relationship, there was an acknowledged need to avoid overstimulation and to recognise cues associated with this:

‘Things like splayed hands and what overstimulation looks like, when they want you to stop [the baby], how to kind of put your hands on them to kind of calm them down if everything’s a bit too much’. [Fatima]

‘He was sneezing, but I didn’t know that sneezing is actually a sign of they’re overstimulated’. [Sue]

Participants mentioned their partners, but it was clear that it was the mother who spent the most time in the neonatal unit. Consequently, there was a worry that fathers could take longer to feel comfortable communicating with their infant.

## Barriers to communication: ‘*A bit of a hindrance*’ [Charlotte]

Although, overall, parents spoke positively about their communication experiences, some did raise barriers. One key factor was the impact of the COVID-19 pandemic which affected both parental presence and facial vision due to mask wearing:

‘Because even things like smiling, the way you position your mouth when you talk, they can’t see that through a mask’. [Sharon].

For some participants, the COVID-19 pandemic had a broader impact as family members (including siblings and grandparents) had not been able to visit the neonatal unit and offer support. Virtual systems were in operation in some neonatal units, meaning that family could watch their infant for a designated time each day, or photographs/videos could be uploaded by nurses to a personal portal – this helped to maintain communication alongside telephone calls.

Participants also spoke of broader issues impacting on communication including a need to leave the unit if another infant was undergoing a medical intervention, lack of screens/curtains when breastfeeding, need to wear headphones (to protect patient confidentiality) during ward rounds that ‘*would go on for hours*’ [Sharon] and mixed messages that they perceived were given by some staff in relation to, for example, when they could hold their infant and for how long. Certainly, communication between parents and staff was pivotal in terms of participants feeling supported and receiving consistent messages in a caring compassionate manner.

## Ongoing need for support at home: ‘*Please can you come and see*’ [Aiyla]

Before going home, an infant was often moved to a different level of neonatal unit, typically a Level 1 (Special Care Baby Unit). This could mean that the same type of support and advice was not available, and parents could find this difficult, especially when nearing discharge home. There was a feeling that, as discharge became closer, there was focus on practicalities such as weight gain. However, at the latter stage of the neonatal unit journey, parents were able to stay with their infant in preparation for transition home and that was perceived to be a:

‘really good bonding time’ [Sharon].

Once home, parents spoke of ongoing communication challenges. There was a feeling that their infant was frustrated and unable to physically do activities that they wanted to do, for example, a 10-month infant (7 months corrected age) was unable to crawl; communication support and follow-up post-discharge home was felt, at times, to be lacking. Several participants mentioned their health visitor but felt that their emphasis was primarily on feeding and weight gain:

‘The health visitor come out for the weight checks and just sort of catch up and things and the community nurses we were liaising with over the phone’. [Maryam]

Advice offered varied, depending on individual expertise; parents felt that this influenced the support they received, it could be limited or very positive:

‘Health visitor is fantastic, she’s really, really, really good’. [Jessica]

Other types of support were mentioned, and some parents referred to ongoing links with a neonatal unit post-discharge (for example, one unit ran a 6-week Baby Massage Programme); this type of contact was clearly very much appreciated.

There were comments on availability of information relating to communicating with premature infants; some resources were accessible via charities such as Tommy’s, The Lullaby Trust and Bliss. Peer support and listening to the stories of others who had similar experiences, was identified by some parents (such as Charlotte and Aiyla). In other instances, practitioners had provided books and leaflets to parents; Fatima felt that much information was equally applicable to any infant and was left wondering why it was not more readily available. Despite this, parents also suggested a need for other types of readily available support/advice at all stages of their journey, such as Apps and social media that were accessible via a Smartphone.

## Discussion

Our study sought to investigate parental understanding of factors that enhance and/or prevent interaction between themselves and their preterm infants within a neonatal unit; this insight was facilitated by undertaking individual interviews. Our research revealed that following the birth of their baby, parents were plunged into the world of a neonatal unit with little or no previous experience of it; they could feel overwhelmed and as though their child was not theirs; unsurprisingly, this made communication with their infant difficult. However, all parents agreed that touch was a fundamental part of their communication with their infant and is something that could be implemented almost immediately; it has long been established that touch can have multifaceted advantages that include the promotion of comfort and the development of bonding (Ramada et al., 2013).

Edney and McHugh (2023) highlight the initial fear that parents can experience at the beginning of a neonatal care journey (something that was echoed by our participants); however, the authors suggest that as the future of an infant becomes more certain, parents are able to more fully engage in a range of activities such as physiotherapy, occupational as well as speech and language therapy – thus emphasising the need to assess parental readiness to participate in different aspects of infant care.

Parents commented positively on all the expert advice they received. Whilst they spoke very highly of the nursing staff and how they encouraged parents to talk to their infant, cuddle them and to undertake skin-to-skin care, there was less emphasis placed on educating parents about verbal communication strategies that could be used; this concurs with earlier work (Maleki et al., 2022). It is possible that nurses may not have had been taught about different communication methods that parents could use; incorporating this into staff training and development as well as having a strong multidisciplinary approach to care could help to address this.

Exploration of the role of parents in communicating with their infant in a neonatal unit is limited; however, Brignoni-Pérez et al. (2021) propose a randomised controlled trial to ascertain the long-term impact of increased speech exposure by mothers on infant development. Other work advocates a need to start speech and language therapy in the neonatal unit at an early stage, suggesting that this should be routinely continued until discharge (Ross et al., 2017). Whilst this is a valid point, as communication advice given to parents on a neonatal unit can be effective for 6 months post-discharge (Milgrom et al., 2013), the need for specific communication support at home was still raised by our participants. Smith et al. (2013) emphasise the need for thorough discharge planning before a family leave the neonatal unit, to encompass access to relevant support services including speech and language therapists. Unfortunately, our parents reported variable experiences when at home with health visitors being the key professional group mentioned. Continuation of advice to parents about language is fundamental since infant development during the first 12 months of life has been found to be linked to parenting practices (and not just an infant’s medical history) (Hass et al., 2022).

It is known that reading to an infant can be beneficial (Saliba et al., 2018) in terms of speech and language development, and it is also something that our participants mentioned. Levesque et al. (2018) in their neonatal unit-based quality improvement initiative provided books written in parents’ own language; they reported that parents enjoyed the reading experience, felt that there were positive benefits and expressed an intention to continue post-discharge. Introducing the concept of parental reading in all neonatal units, with an associated availability of appropriate literature, is a simple and effective strategy to advocate; it is also an activity that other family members, such as siblings and grandparents can be involved in. Whilst maternal singing (mentioned by some of our participants) has been shown to be beneficial for an infant’s physiological status (Filippa et al., 2013), there does not appear to be strong evidence about its positive impact on language development.

Romeo et al. (2023) conducted a study in Boston, USA that focussed on the benefits and barriers to adult-infant communication in the neonatal intensive care unit. The findings indicated that barriers could include equipment, staff having insufficient time and parental anxiety when their baby was very ill; however, whilst our study suggested that some equipment (such as incubators and masks) could be a barrier, we found no indication of the other points. Our research highlighted that another key issue was the time that parents were able to spend in a neonatal unit, this often being restricted by other family/work commitments as well as ongoing COVID-19 restrictions. As found by Romeo et al. (2023), parents in our study were very motivated to overcome barriers and to further develop their communication with their baby; this finding further supports the need to implement tailored strategies to enhance communication, something that has been previously advocated (Mahoney et al., 2017).

In our study, parents commented on how masks limited facial vision, highlighting the impact that this had in terms of communicating with their infant. Use of masks has been previously considered within a neonatal context (Green et al., 2021); Mheidly et al. (2020) explain that the middle and lower parts of the face have a particularly key role in communication. In addition, a correctly applied mask will muffle sound, therefore making it more difficult to interpret and process speech (Mheidly et al., 2020). If face masks are in place in a neonatal unit, these fundamental aspects of communication will not be available to infants, and they are likely to have delayed recognition of different emotions. One potential solution to this is usage of transparent masks; Green et al. (2021) recommend that clear face masks be provided for the parents of babies who are in a neonatal unit for a longer time.

Despite some of the barriers to communication, all parents commented on its positive attributes speaking of how it enhanced bonding with their infant with smiles and facial expressions often calming the baby (findings that concur with Romeo et al., 2023).

## Limitations

Our study used a small data set, a feature of qualitative research (Malterud et al., 2016), but achieving information power. It is recognised that our sample may not have been representative of all parents and regional demographics as participants were recruited via just two neonatal units; therefore, generalisability to a broader population is not feasible, but our findings may provide insight for those in similar situations. In terms of usage of narrative inquiry, it is important to acknowledge that memories can change over time (Kitzinger, 2004) and may be selective; however, the aim of this approach is to hear participants’ perspectives which are ‘*grounded in what is tellable*’ (Atkinson and Coffey, 2003: 118).

Due to the subjective nature of narrative inquiry, it was important to ensure that maximum rigour was applied by using strategies such as external peer review of our research proposal, completion of ‘Good Clinical Practice’ refresher training and having just one team member conduct data collection and another analyse it. The team engaged in ‘reflexivity’ at all stages of the research to facilitate transparency and openness in terms of the power relationship between researchers and participants (which could have potentially influenced interpretation of narratives and associated meanings). These strategies were essential to guard against possible researcher bias and to address any issues with the researcher-participant power relationship.

## Implications for practice

There is a need for clear, consistent, and culturally appropriate communication strategies within neonatal practice. To enable this, health professionals need a greater awareness of how to facilitate communication, supported by tailored education which facilitates their own understanding and also enhances knowledge and confidence in parents. Parents need to be appropriately prepared and equipped to communicate effectively with their premature infant throughout their neonatal care journey and, importantly once they have gone home. We advocate the use of a range of resources that are aimed at enhancing communication between the parent and their preterm baby. There are already some helpful websites (such as Bliss) that families can be directed to; in addition, neonatal units may find it useful to develop their own resources that reflect the needs of their local parent population (for example, informative leaflets in different languages). Most importantly, it is essential that health professionals are aware of a need to facilitate parent–infant communication within neonatal units and post-discharge; a failure to do so may mean that children will experience delayed language development that could impact on their social, cognitive, and emotional development.

## Conclusion

Our study revealed that parents want to communicate with their infants, understand the benefits of it and want to learn as much as possible to enhance their skills. It was clear that the parents trusted the neonatal staff; these personnel are well placed to educate parents and this in turn could positively influence the development of infants, both during their stay in the unit and after discharge. Unfortunately, parents received varying advice and support, both in the neonatal unit and post-discharge, concurring with previous research (Petty et al., 2018, 2019); it is important that a consistent, inclusive and supportive approach (underpinned with appropriate resources) is employed for all families. The benefits to parents are substantive as their anxiety will be reduced, they will bond more readily with their infant as well as develop confidence to identify their baby’s language needs – all of this will serve to enhance the infant’s experience and facilitate their development.
